# Malaria control across borders: quasi-experimental evidence from the Trans-Kunene malaria initiative (TKMI)

**DOI:** 10.1186/s12936-018-2368-4

**Published:** 2018-06-04

**Authors:** Aayush Khadka, Nicole A. Perales, Dorothy J. Wei, Anna D. Gage, Noah Haber, Stéphane Verguet, Bryan Patenaude, Günther Fink

**Affiliations:** 1 Department of Global Health and Population, Harvard T.H. Chan School of Public Health, 655 Huntington Avenue, Boston, MA 02115 USA; 2 University of California Berkeley, Berkeley, CA 94720 USA; 3 Carolina Population Center, University of North Carolina, Chapel Hill, NC 27516 USA; 4 Swiss Tropical and Public Health Institute and University of Basel, Basel, Switzerland

**Keywords:** Malaria, Angola, Namibia, Trans-Kunene malaria initiative, Cross-border, Spillover

## Abstract

**Background:**

The transmission of malaria through population inflows from highly endemic areas with limited control efforts poses major challenges for national malaria control programmes. Several multilateral programmes have been launched in recent years to address cross-border transmission. This study assesses the potential impact of such a programme at the Angolan–Namibian border.

**Methods:**

Community-based malaria prevention programmes involving bed net distribution and behaviour change home visits were rolled-out using a controlled, staggered (stepped wedge) design between May 2014 and July 2016 in a 100 × 40 km corridor along the Angolan–Namibian border. Three rounds of survey data were collected. The primary outcome studied was fever among children under five in the 2 weeks prior to the survey. Multivariable linear and logistic regression models were used to assess overall programme impact and the relative impact of unilateral versus coordinated bilateral intervention programmes.

**Results:**

A total of 3844 child records were analysed. On average, programme rollout reduced the odds of child fever by 54% (aOR: 0.46, 95% CI 0.29 to 0.73) over the intervention period. In Namibia, the programme reduced the odds of fever by 30% in areas without simultaneous Angolan efforts (aOR: 0.70, 95% CI 0.34 to 1.44), and by an additional 62% in areas with simultaneous Angolan programmes. In Angola, the programme was highly effective in areas within 5 km of Namibian programmes (OR: 0.37, 95% CI 0.22 to 0.62), but mostly ineffective in areas closer to inland Angolan areas without concurrent anti-malarial efforts.

**Conclusions:**

The impact of malaria programmes depends on programme efforts in surrounding areas with differential control efforts. Coordinated malaria programming within and across countries will be critical for achieving the vision of a malaria free world.

**Electronic supplementary material:**

The online version of this article (10.1186/s12936-018-2368-4) contains supplementary material, which is available to authorized users.

## Background

Between 2010 and 2016, global malaria incidence declined substantially from 76 to 63 cases per 1000 people at risk [1]. However, the burden of malaria remains high in many areas, with an estimated 445,000 malaria deaths worldwide in 2016, much of which occurred among children under-five [1].

The majority of the current malaria mortality burden is borne by countries in sub-Saharan Africa, where falciparum malaria is most common [1, 2]. Malaria control in many parts of this region has been challenging because of the stability and intensity of malaria transmission [3], emerging insecticide resistance [4], and increasing population movements between high- and low-endemic areas [5–7].

Human mobility across national borders is particularly challenging from a logistical and political perspective. Most malaria programmes are run through national offices, which try to optimize resource allocation and impact within countries, but tend to have limited capacity to affect or implement malaria programmes in areas outside their borders. Even though several cross-border malaria control programmes have been launched in recent years [8–10], little is known regarding the effectiveness of these programmes in general, and evidence on the relative impact of coordinated cross-country efforts remains largely lacking.

This study assesses the effectiveness of a cross-border community-based malaria prevention programme launched at the Angolan–Namibian border in 2012. The programme was launched as part of the Trans-Kunene malaria initiative (TKMI), which was an agreement between the governments of Angola and Namibia to develop, among others, an evidence base for cross-border malaria control strategies and facilitate the sharing of technical and scientific information between the two countries to strengthen malaria transmission and control initiatives. To allow for a rigorous evaluation of the programme, a controlled, staggered (stepped wedge) rollout plan for a 100 × 40 km corridor along the border was developed and implemented between 2014 and 2016. The average programme impact was estimated in a first step, followed by an estimate of the extent to which simultaneous cross-border programming modified the relative impact of the malaria prevention programme.

## Methods

### Study population

The malaria prevention programme was implemented in all villages in a pre-specified 100 × 40 kilometre corridor along the Angolan–Namibian border in the Trans-Kunene region. The Trans-Kunene region comprises of two Angolan provinces—Cunene and Namibe—and three Namibian regions—Kunene, Ohangwena, and Omusati. Target districts for the malaria prevention programme was jointly decided upon by the Ministry of Health in Angola and Namibia. Following this, the intervention corridor was defined by the study team under the assumption that most cross-border infections would occur in this spatial area as movement across the border happens primarily on foot or bicycle. The intervention corridor encompassed Cunene province in Angola, and Ohangwena and Omusati regions in Namibia. Additional file 1 highlights the corridor in the Trans-Kunene region in which the programme was implemented.

The two Angolan provinces in the TKMI represent areas of lower and unstable malaria endemicity in comparison to the rest of the country [11]. In 2015–2016, malaria prevalence among children under-five was reported to be 1% or less in Cunene and Namibe provinces, which was in stark contrast with the neighbouring province of Cuando-Cubango where prevalence was reported to be approximately 38% [12]. Access to preventive measures such as insecticide-treated nets, indoor residual spraying, and malaria treatment was particularly low in the two Angolan provinces in the TKMI.

The three TKMI regions in Namibia have sustained levels of malaria receptivity and account for a majority of malaria cases reported in the country [13, 14]. In contrast, the south of the country is malaria free while the incidence of malaria in the central regions is low [13]. Overall, malaria incidence and mortality has been decreasing in Namibia, which is largely attributable to increased distribution of long-lasting insecticide-treated bed nets (LLITNs), and improved access to malaria treatment [14].

### Community-based malaria prevention intervention

The malaria prevention programme was conducted by community-based volunteers and involved the distribution of LLITNs and behavior change programming. The volunteers did not receive any monetary incentives; instead, they were given an end-of-year food basket, TKMI t-shirts, and light refreshments on the day of net distribution.

For bed nets, community volunteers first compiled a listing of all inhabited structures in programme villages independent of construction type (households). Following this, they delivered and assisted with the installation of rectangular, World Health Organization Pesticides Evaluation Scheme (WHOPES) approved LLITNs in all listed households. Each household received one LLITN per sleeping space. Informal sleeping spaces outside households were not considered during distribution as all families sleep inside their homes at night because of proximity to the land they work in. The distributed LLITNs have an expected lifespan of 3–4 years and have been demonstrated to retain effectiveness up to 20 washes [15]. Systematic reviews have also shown that insecticide treated nets can reduce incidence of *Plasmodium falciparum* malaria episodes by 50% [16].

Behaviour change programming involved community-based volunteers making monthly visits to households to provide guidance on the use and maintenance of LLITNs. In addition, they also provided important malaria prevention information such as strategies for eliminating standing water.

The malaria prevention programme followed a two-phased block rollout schedule between 2014 and 2016. During Phase I, which occurred between 2014 and 2015, all 35 Namibian villages in the intervention corridor as well as 50% of purposely selected areas in Angola received the treatment. As illustrated in Additional file 2, the Angolan corridor was divided into four approximately equally sized zones. For the Phase I rollout, two of these zones were selected to receive the treatment while the remaining zones were used as control areas. There were no buffer zones between these areas as the primary objective of this study was to measure treatment effect spillovers. Instead, rollout was blocked, so that we could directly measure spillovers in Phase I villages based on whether they bordered areas targeted in Phase II.

The rollout of bed nets was centrally controlled and coordinated across both countries. Thus, during Phase I, the delivery and installation of nets occurred at the same time on both sides of the border. Villages that did not receive the treatment in Phase I received it in Phase II, which was conducted between 2015 and 2016. In both phases, most bed nets were distributed and installed before the peak rainfall months of February and March, which roughly coincides with the period of highest malaria burden in the TKMI area.

### Sampling

A random sample of 64 villages in the 100 × 40 km corridor was selected for evaluation prior to programme rollout. The sampling of villages differed by country: in Namibia, a complete list of all 35 village names in the intervention corridor was provided by the local Ministry of Health and a random sample of 26 villages was selected from this sampling frame to get a sample size of at least 250 households. In Angola, the study team conducted an independent listing of all 38 villages in the intervention corridor at the beginning of the study. Due to the larger study team on the Angolan side, all 38 villages were surveyed.

All study villages in the intervention corridor were situated in rural areas, with most villages comprising of 50–100 households. In each of the 64 study villages, a complete household listing was made prior to each survey round. A random sample of 10% of households was then surveyed in each village in each round.

### Data collection

All 64 study villages were surveyed three times over the evaluation period. Additional file 3 illustrates the rollout of the evaluation surveys and the TKMI interventions. The first survey was conducted between May 2014 and July 2014 (baseline) prior to intervention launch. The second survey was conducted after Phase I in September 2015 (midline). The final survey was conducted after the completion of Phase II in July 2016 (endline). Additional file 4 contextualizes the rollout of the surveys in relation to the distribution of bed nets. Most of the questions in the surveys were adapted from the Demographic and Health Surveys, with some additions made by the study team. No formal validation was done for the additional questions.

Each survey consisted of a general household and knowledge module and an under-five child module focused on fever incidence and treatment. Due to budgetary and logistical constraints, malaria status of children under-five was not measured or verified by rapid diagnostic tests (RDTs). A primary respondent, typically a female household member, provided responses to questions in both modules. Of the 2184 surveys attempted over the three rounds, 97% were completed. The most common reasons for non-completion were refusals and temporary unavailability of families.

### Outcome measures

The primary outcome of interest was caregiver-reported child fever. As part of all surveys, respondents were asked to first list all children in their household under the age of five, and then indicate fever episodes and treatment-seeking in the 2 weeks preceding the survey.

The secondary outcomes analysed were malaria knowledge, LLITN utilization among children under-five, and household LLITN ownership. Malaria knowledge was evaluated by asking each respondent 20 true/false questions on the nature, consequences, and treatments for malaria. For the empirical analysis, the percentage of correct responses was computed and converted into a z-score to facilitate interpretation of the estimated coefficients. To determine child LLITN utilization, respondent reports of whether each listed child slept under a LLITN on the night before the survey were used. Household LLITN ownership was defined as the number of LLITNs owned by a household and was determined based on respondent reports as well. The complete English version of the survey questionnaire is included in “Appendix 1”.

### Statistical methods

Summary statistics of key household, respondent, and under-five children characteristics as well as primary and secondary outcomes of interest at baseline were computed. To assess the effectiveness of the malaria prevention programme, data across the three waves were pooled and multivariable linear and logistic regression models that controlled for survey-round fixed effects were estimated. Additionally, the overall implementation period was classified into an immediate period, i.e., the time period within 1 month of intervention implementation, and a 1-year follow-up period. Programme impact was evaluated over both timeframes as well. To determine if programme impact on child morbidity varied by age or gender, sub-group regression analyses were conducted.

**Fig. 1:**
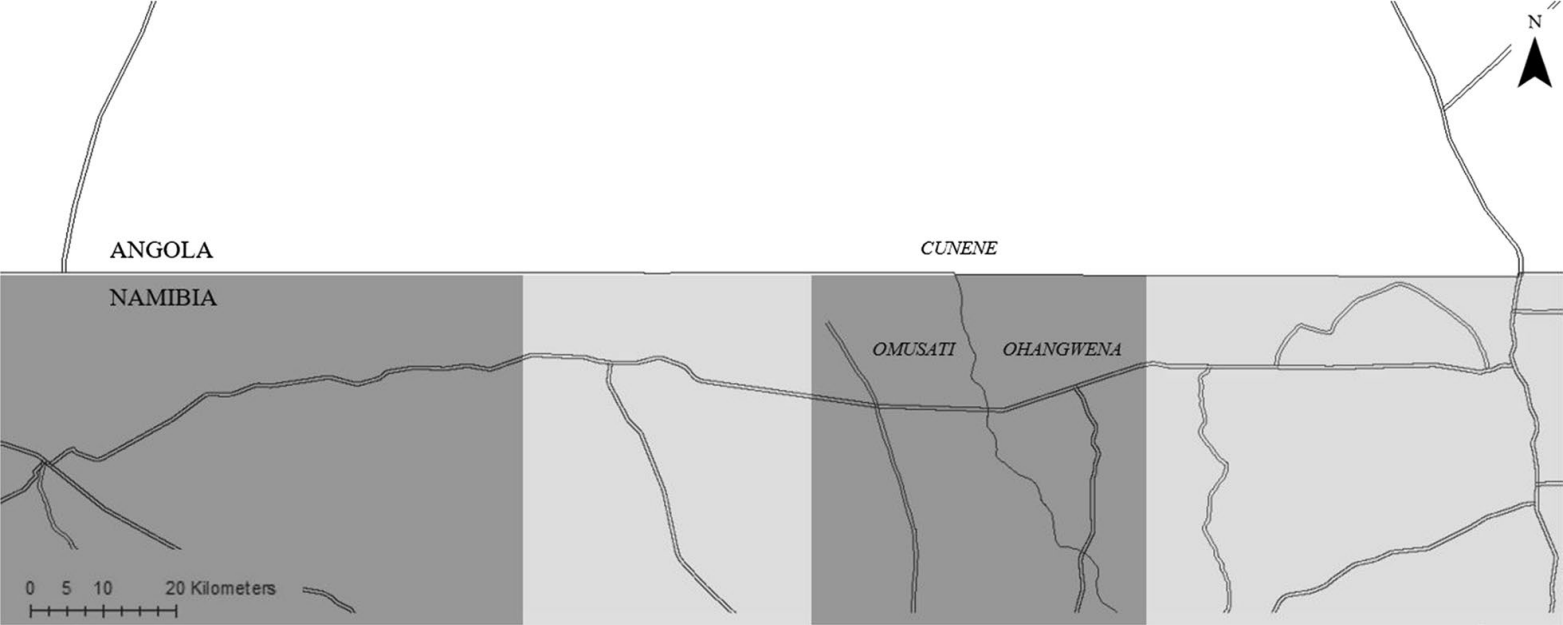
Map demonstrating the grouping of areas in Namibia for the difference-in-difference analysis. The horizontal line in the middle of the map represents the Angola–Namibia border. The labels Cunene, Omusati, and Ohangwena represent the Angolan province and Namibian regions encompassed by the intervention corridor. The double lines represent major road networks in the programme area. Dark gray highlights areas in Namibia that are adjacent to Phase I Angolan areas. Light gray highlights areas in Namibia that are adjacent to Phase II Angolan areas

Two distinct sources of variation were used to assess the extent to which programme impact depends on coordinated cross-border programming (spillover effects). For Namibian villages, variation in programme implementation in Angola during Phase I was exploited to conduct a standard difference-in-differences analysis, interacting Phase I impact in Namibia with Phase I programme activities in Angola. To allow for both linear and multiplicative interactions, linear probability and logistic regression models were estimated. In both models, the main hypothesis tested was that Namibian villages adjacent to Phase I Angolan villages would experience greater programme impact than villages adjacent to Phase II Angolan villages at midline. Figure 1 illustrates this comparison.

For Angolan villages, spatial variation in Phase I treatment status was not available since all Namibian villages received treatment in Phase I. However, since malaria programmes were largely absent north of the TKMI corridor in Angola, programme villages close to Namibia (with extensive malaria control) can be compared with Angolan villages closer to the rest of Angola (with limited programme efforts). To evaluate if distance to the border modified programme impact in Angola, a three-step process was followed: first, kilometre distance between the border and village centroids was computed; second, data were restricted to Phase I Angolan villages; and third, linear and logistic regression models interacted with distance as well as stratified regression models were estimated. For the stratified regressions, the 20 km width of the Angolan half of the corridor was divided into four roughly equally sized strata, and households within 5, 10, 15, or 20 km of the border were considered separately in each model.

To account for correlated outcomes within households and study villages, standard errors in all regression models were estimated using Huber’s cluster-robust variance estimator [17]. This procedure allows correct inference independent of model specification. Missing responses were excluded from the calculation of descriptive statistics and coefficient estimates in the regression models.

### Software

All statistical analyses were conducted in Stata/MP 15.0 [18]. ArcMap 10.3.1 was used to create maps and calculate kilometre distances between villages [19].

### Ethical considerations

This study was approved by the Institutional Review Board at the Harvard T.H. Chan School of Public Health, the Ministry of Health and Social Services, Republic of Namibia, and the Comité de Ética do Ministerio da Saude, Ministério da Saúde, República de Angola (Ethics Committee of the Ministry of Health, Ministry of Health, Republic of Angola).

## Results

A total of 3844 child records across 2126 household surveys were analysed.

### Baseline evaluation

Table 1 presents key baseline characteristics of households, respondents, and under-five children across both countries. At baseline, the average household had nine individuals of whom two were children under 5 years of age. Sixty percent of respondents were female and, on average, were 56 years old and had completed education up to the fourth grade.

**Table 1:** Characteristics of households, survey respondents, and children under-five at baseline (2014)

	Angola and Namibia
Household characteristics	
Number of households	740
Mean household size (N = 739)	9.01 (6.21)
Mean number of children under-five (N = 740)	1.91 (1.58)
Mean number of LLITNs in household (N = 713)	0.77 (1.39)
Mean number of LLITNs used last night (N = 713)	0.60 (1.13)
Mean number of sleeping spaces in households (N = 735)	4.54 (2.56)
Percentage of sleeping spaces covered with bed net on night before the survey (N = 704)	14%
Percentage of households that had visitors from across the border stay the night in the last month (N = 727)	43%
Percentage of households with individuals who have stayed overnight across the border in the last month (N = 732)	44%
Respondent characteristics	
Percentage of female respondents (N = 727)	60%
Mean age of respondents (N = 733)	56 (20)
Mean highest grade achieved in school (N = 715)	4 (4)
Percentage of correct responses to malaria related questions (N = 740)	60%
Under-five children characteristics	
Mean age of under-five children (N = 1311)	2 (1)
Percentage of under-five children who slept under LLITNs last night (N = 1328)	14%
Percentage of under-five children with fever in last 2 weeks (N = 1295)	23%

Standard deviations in parentheses

In terms of study outcomes at baseline: 23% of children under-five were reported to have had fever in the 2 weeks prior to the survey; the average household owned 0.77 LLITNs and had 14% of sleeping spaces covered with nets on the night before the survey; 14% of children under-five were reported to have slept under a LLITN on the night before the survey; finally, respondents correctly answered 60% of the malaria knowledge questions. Additional file 5: Table S1 shows heterogeneity in outcomes at baseline by village.

### Programme impact evaluation

Table 2 presents results from multivariable logistic regression models estimating average programme impact on child fever. Column 1 shows the overall estimated impact; columns 2–5 show estimated impact by age and gender.

**Table 2:** Programme impact on respondent-reported fever among children under-five

Outcome Sample	Fever episode in 2 weeks prior to survey				
	All children under-five	Age < 2 years	Age ≥ 2 years	Male children	Female children
	(1)	(2)	(3)	(4)	(5)
Treated	0.464*** (0.294 to 0.731)	0.387*** (0.230 to 0.649)	0.531** (0.304 to 0.929)	0.649 (0.340 to 1.239)	0.332*** (0.199 to 0.553)
Constant	0.292*** (0.242 to 0.353)	0.415*** (0.324 to 0.532)	0.243*** (0.193 to 0.305)	0.312*** (0.249 to 0.390)	0.285*** (0.225 to 0.360)
H_0_: equal impact^a^	NA	p = 0.08		p = 0.462	
Observations	3750	1049	2612	1898	1783

Multivariable logistic regression results showing average programme impact on under-five child fever (Column 1), fever episodes among children under 2 years (Column 2), fever episodes among children between 2 and 4 years (Column 3), fever episode among male children (Column 4), and fever episode among female children (Column 5). All models control for survey-round fixed effects

95% confidence intervals shown in parentheses are based on Huber’s cluster robust variance estimator

*** p < 0.01, ** p < 0.05, * p < 0.1

^a^p-values based on a pooled linear model with an interaction term between age and treatment (Columns 2 and 3) and sex and treatment (Columns 4 and 5), respectively

The programme reduced the odds of fever by 54% (aOR: 0.46, 95% CI 0.29 to 0.73) over the 2 year intervention period. Among children under 2 years, the programme reduced the odds of fever by 71% (aOR: 0.39, 95% CI 0.23 to 0.65). In comparison, among children over 2 years, the programme reduced the odds of fever by 47% (aOR: 0.53, 95% CI 0.30 to 0.93). However, the difference in treatment effect between the two age groups was not statistically significant. Programme impact also appeared to be larger among female children by 67% (aOR: 0.33, 95% CI 0.20 to 0.55); once again, treatment effect differences by gender were not statistically significant.

Table 3 presents multivariable linear regression results estimating programme impact on secondary outcomes of interest. On average, the intervention increased the likelihood of LLITN utilization among children under-five by 57% points (β = 0.57, 95% CI 0.46 to 0.67), the average number of LLITNs owned by households by 4.36 (95% CI 3.46 to 4.96), and the average malaria knowledge scores by 0.35 standard deviations (95% CI 0.03 to 0.67).

**Table 3:** Programme impact on secondary outcomes

Outcome	(1)	(2)	(3)
	Under-five child slept under LLITN on the night prior to the survey	Household LLITN ownership	Knowledge score (z-score)
Treated	0.568*** (0.464 to 0.672)	4.357*** (3.761 to 4.953)	0.352** (0.0335 to 0.670)
Constant	0.144*** (0.0934 to 0.194)	0.769*** (0.551 to 0.986)	− 0.279*** (− 0.434 to − 0.124)
Observations	3788	2093	2126
R-squared	0.347	0.338	0.065

Multivariable linear regression results showing average programme impact on LLITN utilization among children under-five (Column 1), household LLITN ownership (Column 2), and malaria knowledge z-scores (Column 3). All models were estimated using Ordinary Least Squares regression models. Although not displayed, all models control for survey-round fixed effects. 95% confidence intervals shown in parentheses are based on Huber’s cluster robust variance estimator

*** p < 0.01, ** p < 0.05, * p < 0.1

Additional file 6 disaggregates the programme’s impact on the primary and secondary outcomes of interest into the immediate term and over a 1-year follow-up period. The immediate impact of the programme closely mirrored the overall programme impact across all four outcomes of interest, while second year effects were mixed.

Table 4 shows the first set of spillover results using data from Namibia. In comparison to baseline, decline in fever prevalence at midline among Namibian villages without complementary Phase I malaria programme efforts in Angola was not statistically significant. In contrast, Namibian villages exposed to coordinated Angolan programmes experienced an additional 17% points (β = − 0.17, 95% CI −0.337 to − 0.003) decrease in child fever at midline in comparison to unexposed villages. This corresponds to an approximately 62% additional reduction in the odds of fever.

**Table 4:** Difference-in-differences analysis assessing treatment effect modification among Namibian villages between baseline and midline

Outcome	Fever episode in 2 weeks prior to survey	
	Linear probability model	Logistic model
	(1)	(2)
Post^a^	− 0.053 (− 0.165 to 0.059)	0.698 (0.338 to 1.444)
Post × complementary angolan programme effort	− 0.170** (− 0.337 to − 0.003)	0.377 (0.135 to 1.049)
Observations	706	706
R-squared	0.039	

Multivariable regression results from a difference-in-differences analysis. Column 1 and Column 2 show results based on a linear probability model and logistic regression model respectively. Although not shown in the table, the models control for an indicator of Namibian villages adjacent to Phase I Angolan areas at baseline. Constant terms from the two models are also not shown in the table. Data for this analysis is restricted to Namibian households surveyed at baseline and midline

95% confidence intervals shown in parentheses are based on Huber’s cluster robust variance estimator

*** p < 0.01, ** p < 0.05, * p < 0.1

^a^All Namibian villages were treated between baseline and midline, which means that the post indicator captures both time and treatment effects

Additional file 7 shows unadjusted village level mean differences in child fever prevalence between baseline and midline for Namibian villages disaggregated by areas exposed and unexposed to coordinated cross-border programme efforts. While these results are primarily descriptive, villages exposed to coordinated efforts in Angola appear to have had a higher likelihood of experiencing significant fever prevalence declines in comparison to villages unexposed to coordinated efforts.

Table 5 shows results from the second spillover test. In terms of Phase I treatment effects in Angola, the intervention reduced the odds of fever by 55% (aOR: 0.45, 95% CI 0.24 to 0.81) on average at the border. For every kilometre increase in distance from the border, the additional change in treatment effect on child fever is reduced by approximately 8% (aOR: 1.08, 95% CI 1.03 to 1.13).

**Table 5:** Interaction between programme impact in Angola and distance to Namibian border

Outcome	Fever episode in 2 weeks prior to survey	
	Linear probability model	Logistic regression model
	(1)	(2)
Treated (midline)	− 0.112** (− 0.200 to − 0.0233)	0.447*** (0.244 to 0.817)
Distance	− 0.000580 (− 0.00802 to 0.00686)	0.996 (0.955 to 1.040)
Treated* distance	0.0111*** (0.00384 to 0.0184)	1.078*** (1.032 to 1.127)
Baseline	0.211*** (0.121 to 0.302)	0.268*** (0.160 to 0.449)
Observations	1469	1469
R-squared	0.013	

Multivariable regression results from analysis for treatment effect modification in Angola. Results based on linear probability model are shown in Column 1. Results based on logistic regression model are shown in Column 2. 95% confidence intervals shown in parentheses are based on Huber’s cluster robust variance estimator. Sample is restricted to Angolan villages receiving treatment between baseline and midline

*** p < 0.01, ** p < 0.05, * p < 0.1

Stratified regression results presented in Table 6 further illustrate these rather large interaction effects. The intervention was highly effective in areas close to the Namibian border with an estimated odds reduction of 63% (OR: 0.37, 95% CI 0.22 to 0.62) within areas 5 km of the border. However, the programme did not have any significant impact in areas more distant from Namibia (i.e., closer to non-treated Angolan areas). For areas more than 15 km from the Namibian border (and, therefore, right at the border of non-treated Angolan villages), fever prevalence increased between baseline and midline despite programme rollout.

**Table 6:** Programme impact in Angola, stratified by distance to Namibian border

Outcome	Fever episode in 2 weeks prior to survey			
Sample	(1)	(2)	(3)	(4)
	< 5 km of the Namibia border	5–10 km from the border	10–15 km from the border	> 15 km from the border
Treated	0.368*** (0.217 to 0.624)	0.962 (0.342 to 2.704)	1.156 (0.691 to 1.934)	1.835* (0.976 to 3.450)
Constant	0.299*** (0.167 to 0.535)	0.203*** (0.101 to 0.407)	0.290*** (0.252 to 0.334)	0.245*** (0.105 to 0.572)
Observations	461	439	445	124

Logistic regression analysis demonstrating treatment effect on child morbidity within 5 km of the Angola–Namibia border (Column 1), between 5 and 10 km of the border (Column 2), between 10 and 15 km of the border (Column 3), and beyond 15 km of the border (Column 4). 95% confidence intervals shown in parentheses are based on Huber’s cluster robust variance estimator. Sample is restricted to Angolan villages receiving treatment between baseline and midline

*** p < 0.01, ** p < 0.05, * p < 0.1

## Discussion

The results presented in this paper have yielded two main insights. First, community-based malaria prevention programmes appear to remain highly effective in reducing child morbidity, at least in areas with seasonal malaria and relatively little intervention coverage in the recent past. This study shows that over the 2-year intervention period, a relatively simple community volunteer-based prevention programme which distributed LLITNs and had monthly behavior change home visits before the peak rainfall months of February and March reduced the odds of child fever by more than 50% overall.

Second, and more importantly, this study shows that the effectiveness of community-based malaria programmes strongly depends on concurrent efforts made in neighbouring areas. The estimates presented in this paper suggest that Namibian areas at the border benefitting both from local programmes and simultaneously implemented programmes in adjacent Angolan areas experienced more than twice the reductions in fever than areas benefitting from local programmes only. The results are starker for Angola, where there were impressive reductions in fever prevalence in areas close to Namibian villages benefitting from programmes, but no health improvements at all in areas closer to Angolan villages not benefitting from any programme.

These rather large local spillovers make sense from a biological and public health perspective as they likely reflect both vector and human population movements between villages in the programme corridor. At baseline, 44% of households reported having had at least one individual stay on the other side of the border in the month before the survey. Similarly, 43% of households at baseline reported having had guests from the other country during the same time period. Such a degree of cross-border mobility seems natural given the absence of major barriers as well as the shared history and culture on both sides of the border. The results from this study may, therefore, be relevant to many other border regions which likely demonstrate similarly high levels of cross-border human population movement.

A high degree of cross-border human mobility does, however, pose a major challenge to malaria programming since neighbouring country’s preferences are unlikely to align. The Angola-Namibia setting is almost ideal to illustrate this: while Namibia has almost eliminated malaria in most parts of the country other than the regions bordering Angola and Zambia, malaria is still endemic in most parts of Angola, and is particularly common in the Northern parts of the country. From an Angolan perspective, the burden of malaria is relatively low in the South; thus, such areas are not a primary target of Angolan efforts. The opposite is true for Namibia, which focuses most of its efforts on the northern regions of the country.

The results presented in this study suggest optimal effectiveness may only be reached if efforts are coordinated across the border. Since countries with a high malaria burden generally have little incentive to focus on areas bordering low transmission countries, coordinated cross-country efforts will likely have to be developed and supported by external stakeholders. A general switch in focus towards regional rather than national efforts, therefore, seems advisable from a political and donor perspective, even though such regional efforts would undoubtedly require increased coordination and monitoring efforts to ensure overall accountability. Encouragingly, there has already been an increasing focus on regional programming to decrease cross-border transmission, especially in Southern Africa: for instance, the Elimination 8 (E8) secretariat has supported projects to increase access to testing and treatment through malaria surveillance posts at strategic locations at the borders shared by the eight E8 member states.

This study has several limitations. The most immediate and obvious limitation is that it does not use directly confirmed cases of malaria among children as the primary outcome. Malaria cases were not recorded as it was infeasible within the study budget to license community health volunteers in using RDTs and have them visit households with high frequency. While fever is the most common manifestation of malaria, it can be a symptom of many other illnesses as well [11]. It is also possible that subjects over- or underreported fever. If such incorrect reporting is correlated with programme rollout, estimated results may be biased. In terms of the main results presented in this paper, potential social desirability biases are more likely to apply to the overall programme impact estimates since the provision of (highly appreciated) nets may induce grateful households to over-report positive health outcomes. In terms of the spillover estimates, all households received the same interventions and should thus be equally prone to such biases unless they directly take the relative treatment status of neighboring villages into consideration in their responses; however, this seems unlikely.

A second limitation of the analysis is that the rollout of the treatment was not randomized at the village or cluster level, but rather followed a two-phase blocked rollout schedule. Exploring this rollout schedule will yield unbiased impact results as long as treated and control villages would have experienced the same trajectories in the outcomes in the absence of treatment. While this assumption cannot be tested directly, it is likely to hold since no other major health programmes were run in the intervention corridor during the study period. Furthermore, given the large effects found and the somewhat arbitrary blocking of villages, major confounding biases seem somewhat unlikely overall.

Given the data, it is also not possible to directly distinguish the impact of LLITNs from the impact of behavior change oriented home visits on morbidity. Similarly, the data do not allow for evaluating the relative effectiveness of guidance on using and maintaining LLITNs versus provision of malaria prevention information on changes in morbidity. The combination of the two interventions may at least partially explain the relatively large impacts seen in this study compared to the previous literature [16]. Further research will be needed to disentangle the impact of the two intervention components.

## Conclusions

The World Health Assembly and Roll Back Malaria campaign have endorsed the vision of a malaria free world, aiming for a 90% reduction in malaria incidence and mortality by 2030 [4, 20]. The WHO Global Technical Strategy identifies cross-border collaboration as a key mechanism by which the vision of a malaria free world can be made a reality [20]. The results presented in this paper provide strong evidence for the importance of increased cross-national collaboration and coordination of anti-malaria efforts, particularly in settings with high levels of cross-border human mobility. While more research is needed to understand the relative contribution of different cross-border interventions, this study shows that even simple interventions can be extremely effective in reducing child morbidity.

### Additional files

**Additional file 1: Figure S1.** Location of TKMI programme area. The main map shows Angola (dark gray) and Namibia (light gray) and depicts provincial boundaries within each country. The numerical labels indicate the administrative areas in which the TKMI program was implemented: (1) = Namibe; (2) = Cunene; (3) = Kunene; (4) = Omusati; (5) = Ohangwena. The black rectangle within the main map shows the region being demonstrated in the inset map. The inset map shows the TKMI programme area and the crosses show the 64 villages selected for the evaluation of the malaria control programme. The horizontal line in the middle of the inset map represents the Angola–Namibia border.

**Additional file 2: Figure S2.** Programme roll-out map. Maps illustrating the coverage of the programme at each survey round. Panel (a) highlights the boundaries of the intervention corridor and shows how none of the areas had received any treatment at baseline. Panel (b) highlights areas in Angola and Namibia that received treatment during Phase I. Panel (c) shows that all programme areas had received the intervention by endline. The horizontal line across the middle of the map represents the Angola–Namibia border. The labels Cunene, Omusati, and Ohangwena represent the Angolan province and Namibian regions encompassed by the intervention corridor. The double lines represent major road networks in the programme area.

**Additional file 3: Figure S3.** Programme and evaluation implementation schedule. Figure depicting the rollout of the TKMI evaluation surveys and the interventions.

**Additional file 4: Figure S4.** Gantt Chart describing timing of programme rollout and evaluation. Gantt Chart describing timing of programme rollout and evaluation.

**Additional file 5: Table S1.** Baseline child fever, LLITN ownership, usage, and malaria knowledge levels. Baseline statistics on a per village basis disaggregated by country.

**Additional file 6: Table S2.** Disaggregating TKMI impact into immediate and follow-up effect. Programme impact disaggregated by immediate and 1-year follow-up period.

**Additional file 7: Figure S5.** Forest plots illustrating unadjusted mean difference in fever prevalence between baseline and midline among Namibian villages. **a** presents mean differences in villages exposed to coordinated cross-border efforts while **b** presents mean differences for villages unexposed to coordinated efforts.

## Appendix 1

Questionnaire
