# Correction to: Malaria control across borders: quasi-experimental evidence from the Trans-Kunene malaria initiative (TKMI)

**DOI:** 10.1186/s12936-019-2997-2

**Published:** 2019-11-14

**Authors:** Aayush Khadka, Nicole A. Perales, Dorothy J. Wei, Anna D. Gage, Noah Haber, Stéphane Verguet, Bryan Patenaude, Günther Fink

**Affiliations:** 1 Department of Global Health and Population, Harvard T.H. Chan School of Public Health, 655 Huntington Avenue, Boston, MA 02115 USA; 2 University of California Berkeley, Berkeley, CA 94720 USA; 3 Carolina Population Center, University of North Carolina, Chapel Hill, NC 27516 USA; 4 Swiss Tropical and Public Health Institute and University of Basel, Basel, Switzerland

## Correction to: Malar J (2018) 17:224 10.1186/s12936-018-2368-4

Following publication of the original article [1], the authors flagged an error in Addition file 6.

The error is in the coefficient estimates presented in Column 2 of the table; exponentiated values are presented instead of standard OLS coefficients.

Please therefore now be referred to the corrected version of Additional file 6 contained in this article.

The results and analysis of the article are not affected by this error.

The authors apologize for any inconvenience caused.

## Supplementary information

**Additional file 6: Table S2.** Disaggregating TKMI impact into immediate and follow-up effect. Programme impact disaggregated by immediate and 1-year follow-up period.
