# Supplementary figures and images for: Malaria control across borders: quasi-experimental evidence from the Trans-Kunene malaria initiative (TKMI)
Source: Malar J. 2018 Jun 4;17:224. doi: 10.1186/s12936-018-2368-4 (PMC5987525; doi:10.1186/s12936-018-2368-4)

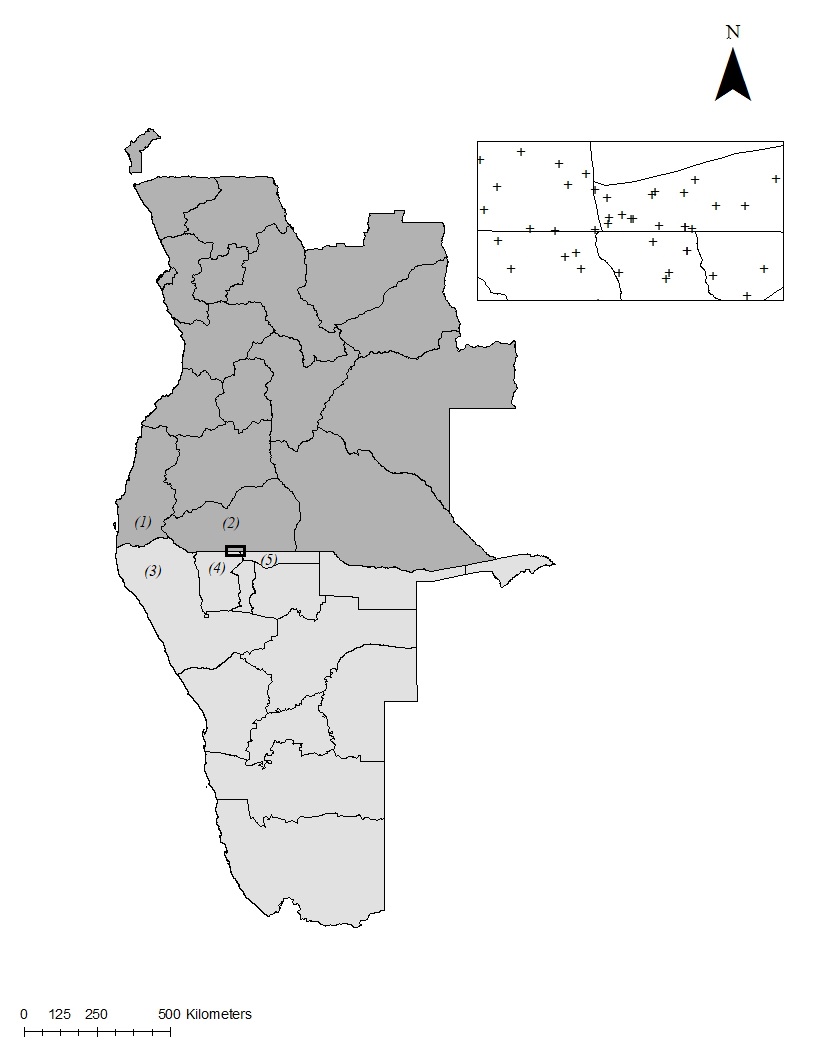

Supplement: Supplementary file 1 — Additional file 1: Figure S1. Location of TKMI programme area. The main map shows Angola (dark gray) and Namibia (light gray) and depicts provincial boundaries within each country. The numerical labels indicate the administrative areas in which the TKMI program was implemented: (1) = Namibe; (2) = Cunene; (3) = Kunene; (4) = Omusati; (5) = Ohangwena. The black rectangle within the main map shows the region being demonstrated in the inset map. The inset map shows the TKMI programme area and the crosses show the 64 villages selected for the evaluation of the malaria control programme. The horizontal line in the middle of the inset map represents the Angola–Namibia border. [file 12936_2018_2368_MOESM1_ESM.jpg]

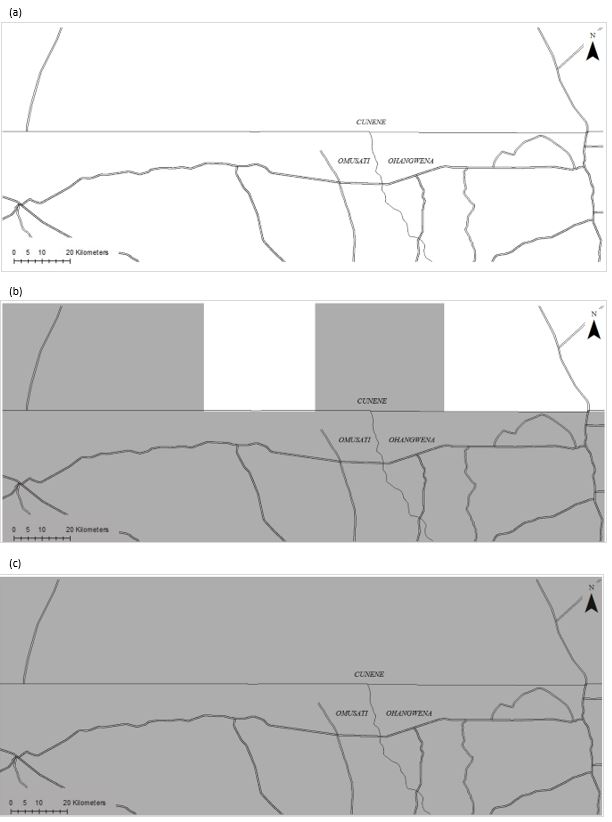

Supplement: Supplementary file 2 — Additional file 2: Figure S2. Programme roll-out map. Maps illustrating the coverage of the programme at each survey round. Panel (a) highlights the boundaries of the intervention corridor and shows how none of the areas had received any treatment at baseline. Panel (b) highlights areas in Angola and Namibia that received treatment during Phase I. Panel (c) shows that all programme areas had received the intervention by endline. The horizontal line across the middle of the map represents the Angola–Namibia border. The labels Cunene, Omusati, and Ohangwena represent the Angolan province and Namibian regions encompassed by the intervention corridor. The double lines represent major road networks in the programme area. [file 12936_2018_2368_MOESM2_ESM.jpg]

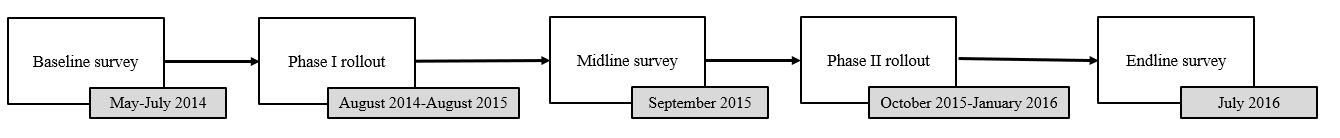

Supplement: Supplementary file 3 — Additional file 3: Figure S3. Programme and evaluation implementation schedule. Figure depicting the rollout of the TKMI evaluation surveys and the interventions. [file 12936_2018_2368_MOESM3_ESM.jpg]

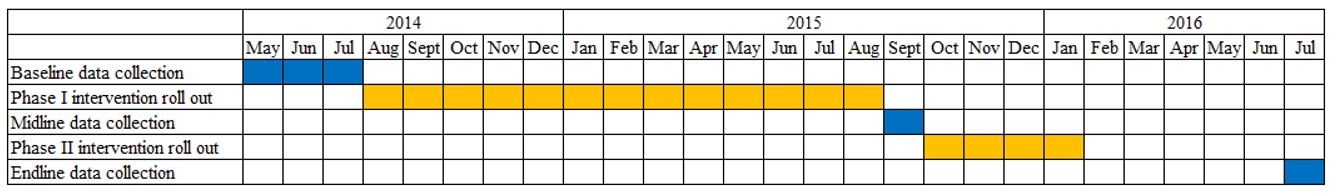

Supplement: Supplementary file 4 — Additional file 4: Figure S4. Gantt Chart describing timing of programme rollout and evaluation. Gantt Chart describing timing of programme rollout and evaluation. [file 12936_2018_2368_MOESM4_ESM.jpg]

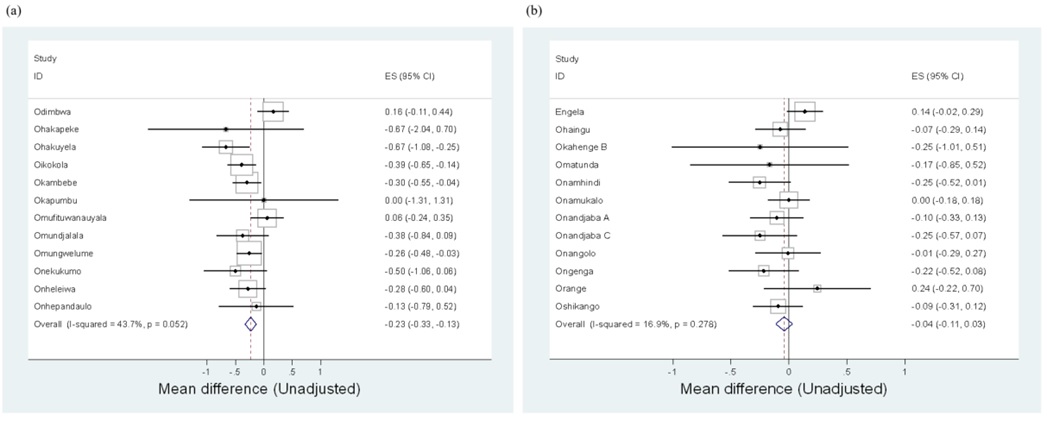

Supplement: Supplementary file 7 — Additional file 7: Figure S5. Forest plots illustrating unadjusted mean difference in fever prevalence between baseline and midline among Namibian villages. a presents mean differences in villages exposed to coordinated cross-border efforts while b presents mean differences for villages unexposed to coordinated efforts. [file 12936_2018_2368_MOESM7_ESM.jpg]
