# Supplementary material for: Malaria control across borders: quasi-experimental evidence from the Trans-Kunene malaria initiative (TKMI)
Source: Malar J. 2018 Jun 4;17:224. doi: 10.1186/s12936-018-2368-4 (PMC5987525; doi:10.1186/s12936-018-2368-4)
Supplement: Supplementary file 5 — Additional file 5: Table S1. Baseline child fever, LLITN ownership, usage, and malaria knowledge levels. Baseline statistics on a per village basis disaggregated by country. [file 12936_2018_2368_MOESM5_ESM.docx]

# **Additional file 5: Baseline child fever, LLITN ownership, usage, and malaria knowledge levels**

Baseline statistics on a per village basis disaggregated by country.

|  |  | Child fever | | LLITN ownership | | Under-five LLITN usage | | Knowledge | |
| --- | --- | --- | --- | --- | --- | --- | --- | --- | --- |
| Country | **Community/Village** | **N** | **%** | **N** | **Mean** | **N** | **Mean** | **N** | **%** |
| Angola | Eembadja | 25 | 12 | 9 | 0.00 | 30 | 0% | 9 | 52 |
|  | Eenghala | 25 | 20 | 17 | 0.06 | 26 | 0% | 17 | 50 |
|  | Eno La Shonena | 10 | 10 | 4 | 0.75 | 10 | 10% | 4 | 70 |
|  | Epumbulile | 9 | 33 | 8 | 0.38 | 9 | 33% | 8 | 52 |
|  | Odila Sede | 20 | 20 | 14 | 0.07 | 20 | 5% | 14 | 63 |
|  | Odishana | 49 | 12 | 19 | 0.21 | 49 | 4% | 19 | 60 |
|  | Ofenda | 35 | 9 | 11 | 0.27 | 35 | 9% | 17 | 55 |
|  | Ofine | 13 | 15 | 8 | 0.38 | 13 | 0% | 8 | 57 |
|  | Ohakadu | 15 | 0 | 10 | 0.10 | 15 | 0% | 10 | 54 |
|  | Ohamanghono | 17 | 12 | 9 | 0.00 | 17 | 0% | 9 | 71 |
|  | Ohamufi | 18 | 39 | 12 | 0.50 | 18 | 22% | 12 | 60 |
|  | Ohanamulu | 25 | 24 | 12 | 1.17 | 25 | 40% | 12 | 50 |
|  | Oiheke | 34 | 18 | 12 | 0.67 | 33 | 0% | 18 | 59 |
|  | Okadu | 11 | 64 | 1 | 2.00 | 11 | 9% | 6 | 51 |
|  | Okahoho | - | - | - | - | - | - | - | - |
|  | Okaku Sede | 22 | 32 | 9 | 0.22 | 22 | 0% | 9 | 48 |
|  | Okanghudi | 8 | 0 | 3 | 2.67 | 8 | 38% | 3 | 66 |
|  | Olutula 2 | 35 | 14 | 18 | 0.00 | 37 | 0% | 18 | 65 |
|  | Omukwa wa Shindyala | 17 | 18 | 11 | 0.36 | 17 | 0% | 11 | 57 |
|  | Omulunga | 18 | 33 | 14 | 0.79 | 18 | 22% | 14 | 57 |
|  | Omupepo | 17 | 12 | 10 | 0.10 | 17 | 0% | 10 | 57 |
|  | Omutitu Wamautano | 38 | 18 | 11 | 0.00 | 39 | 0% | 11 | 58 |
|  | Onanbungu | 24 | 25 | 17 | 0.82 | 25 | 20% | 17 | 59 |
|  | Onanghombo | 23 | 9 | 11 | 0.55 | 24 | 13% | 14 | 66 |
|  | Onanghulo | 29 | 38 | 23 | 0.39 | 29 | 10% | 23 | 58 |
|  | Onangwena | 6 | 33 | 5 | 0.00 | 6 | 0% | 7 | 70 |
|  | Onashalamba | 18 | 11 | 11 | 0.82 | 18 | 50% | 11 | 64 |
|  | Ondago | 58 | 34 | 26 | 2.31 | 59 | 37% | 26 | 63 |
|  | Ondjengo | 21 | 19 | 15 | 0.53 | 21 | 5% | 15 | 53 |
|  | Ondombe Yakaoli | 45 | 38 | 14 | 0.00 | 45 | 2% | 14 | 65 |
|  | Onghoshi | 9 | 11 | 8 | 1.00 | 9 | 56% | 8 | 59 |
|  | Onghuiyu | 39 | 3 | 16 | 0.06 | 39 | 3% | 16 | 59 |
|  | Ongode Sede | 19 | 21 | 10 | 0.20 | 19 | 11% | 10 | 48 |
|  | Onhedeyeti | 8 | 38 | 7 | 0.57 | 8 | 0% | 7 | 67 |
|  | Oshaixwanda | 108 | 24 | 39 | 0.21 | 116 | 3% | 39 | 57 |
|  | Oshikexweme | 37 | 22 | 20 | 0.20 | 37 | 8% | 20 | 42 |
|  | Oshilhamga | 32 | 22 | 17 | 0.59 | 32 | 22% | 17 | 53 |
|  | Ounonge | 5 | 0 | 7 | 0.00 | 5 | 0% | 7 | 60 |
| Namibia | Engela | 51 | 12 | 41 | 1.17 | 53 | 43% | 42 | 71 |
|  | Odimbwa | 15 | 7 | 9 | 0.44 | 15 | 27% | 9 | 57 |
|  | Ohaingu | 23 | 22 | 11 | 0.64 | 24 | 17% | 11 | 63 |
|  | Ohakapeke | 3 | 67 | 4 | 0.75 | 3 | 0% | 4 | 66 |
|  | Ohakuyela | 3 | 67 | 5 | 0.40 | 7 | 0% | 5 | 58 |
|  | Oikokola | 23 | 39 | 12 | 0.00 | 23 | 0% | 12 | 61 |
|  | Okahenge B | 4 | 25 | 3 | 0.00 | 4 | 0% | 3 | 70 |
|  | Okaku Ka Imalwa | 2 | 0 | 2 | 0.50 | 2 | 50% | 2 | 55 |
|  | Okambebe | 24 | 46 | 17 | 1.29 | 24 | 29% | 17 | 59 |
|  | Okapumbu | 3 | 33 | 2 | 1.00 | 3 | 33% | 2 | 50 |
|  | Omatunda | 6 | 50 | 3 | 0.67 | 9 | 0% | 3 | 67 |
|  | Omufituwanauyala | 16 | 13 | 8 | 2.25 | 16 | 13% | 8 | 61 |
|  | Omundjalala | 10 | 50 | 4 | 3.75 | 10 | 0% | 4 | 76 |
|  | Omungwelume | 21 | 33 | 24 | 1.42 | 21 | 29% | 25 | 75 |
|  | Onamhindi | 13 | 31 | 10 | 2.00 | 13 | 23% | 11 | 72 |
|  | Onamukalo | 16 | 6 | 8 | 2.13 | 16 | 31% | 8 | 70 |
|  | Onandjaba A | 18 | 17 | 8 | 3.13 | 19 | 58% | 8 | 64 |
|  | Onandjaba B | 2 | 100 | 3 | 0.67 | 2 | 0% | 4 | 60 |
|  | Onandjaba C | 4 | 25 | 6 | 1.17 | 7 | 57% | 6 | 62 |
|  | Onangolo | 10 | 10 | 7 | 1.00 | 10 | 20% | 7 | 63 |
|  | Onekukumo | 6 | 50 | 4 | 1.75 | 6 | 0% | 4 | 57 |
|  | Ongenga | 16 | 38 | 16 | 1.50 | 16 | 25% | 17 | 68 |
|  | Onheleiwa | 22 | 36 | 10 | 2.40 | 22 | 36% | 10 | 54 |
|  | Onhepandaulo | 3 | 33 | 6 | 0.33 | 3 | 0% | 6 | 59 |
|  | Orange | 10 | 20 | 7 | 1.29 | 10 | 20% | 7 | 61 |
|  | Oshikango | 29 | 24 | 15 | 1.87 | 29 | 21% | 15 | 69 |
