# Supplementary material for: Malaria control across borders: quasi-experimental evidence from the Trans-Kunene malaria initiative (TKMI)
Source: Malar J. 2018 Jun 4;17:224. doi: 10.1186/s12936-018-2368-4 (PMC5987525; doi:10.1186/s12936-018-2368-4)
Supplement: Supplementary file 6 — Additional file 6: Table S2. Disaggregating TKMI impact into immediate and follow-up effect. Programme impact disaggregated by immediate and 1-year follow-up period. [file 12936_2018_2368_MOESM6_ESM.docx]

# **Additional file 6: Disaggregating TKMI impact into immediate and follow-up effect**

Programme impact disaggregated by immediate and one-year follow-up period

|  | (1) | (2) | (3) | (4) |
| --- | --- | --- | --- | --- |
| VARIABLES | Fever episode in two weeks prior to survey among children under-five | Under-five child slept under LLITN on the night prior to the survey | Average LLITN ownership | Knowledge score (z-score) |
|  |  |  |  |  |
| Treated_(t=1)_ | 0.464*** | 1.765*** | 4.357*** | 0.352** |
|  | (0.294 - 0.731) | (1.591 - 1.959) | (3.761 - 4.953) | (0.0334 - 0.670) |
| Treated_(t=2)_ | 0.515* | 1.592*** | 4.670*** | 0.772** |
|  | (0.242 - 1.095) | (1.331 - 1.904) | (3.496 - 5.845) | (0.182 - 1.362) |
| Constant | 0.292*** | 1.155*** | 0.769*** | -0.279*** |
|  | (0.242 - 0.353) | (1.098 - 1.214) | (0.551 - 0.986) | (-0.434 - 0.124) |
|  |  |  |  |  |
| Observations | 3,750 | 3,788 | 2,093 | 2,126 |
| R-squared |  | 0.349 | 0.339 | 0.072 |
| *Notes:* Multivariable regression results showing the TKMI’s impact disaggregated by time period on under-five children fever (Column 1), LLITN utilization among children under-five (Column 2), LLITN ownership (Column 3), and malaria knowledge of respondents (Column 4). Column 1 presents results from a logistic regression model. Columns 2-4 present results from linear regression models. Although not displayed, all models control for survey-round fixed effects. 95% confidence intervals are show in parentheses and are based on Huber’s cluster robust variance estimator. | | | | |
| *** p<0.01, ** p<0.05, * p<0.1 | |  |  |  |
