# Supplementary material for: Correction to: Malaria control across borders: quasi-experimental evidence from the Trans-Kunene malaria initiative (TKMI)
Source: Malar J. 2019 Nov 14;18:365. doi: 10.1186/s12936-019-2997-2 (PMC6854627; doi:10.1186/s12936-019-2997-2)
Supplement: Supplementary file 1 — Additional file 6: Table S2. Disaggregating TKMI impact into immediate and follow-up effect. Programme impact disaggregated by immediate and 1-year follow-up period. [file 12936_2019_2997_MOESM6_ESM.pdf]

Additional file 6: Disaggregating TKMI impact into immediate and follow-up effect

Programme impact disaggregated by immediate and one-year follow-up period

VARIABLES	(1)<br>Fever episode in two weeks prior to survey among children under-five	(2)<br>Under-five child slept under LLITN on the night prior to the survey	(3)<br>Average LLITN ownership	(4)<br>Knowledge score (z-score)
$Treated_{(t=1)}$	0.464***	0.568***	4.357***	0.352**
	(0.294 - 0.731)	(0.464 - 0.672)	(3.761 - 4.953)	(0.0334 - 0.670)
$Treated_{(t=2)}$	0.515*	0.465***	4.670***	0.772**
	(0.242 - 1.095)	(0.286 - 0.644)	(3.496 - 5.845)	(0.182 - 1.362)
Constant	0.292***	0.144***	0.769***	-0.279***
	(0.242 - 0.353)	(0.093 - 0.194)	(0.551 - 0.986)	(-0.434 - 0.124)
Observations	3,750	3,788	2,093	2,126
R-squared		0.349	0.339	0.072

Notes: Multivariable regression results showing the TKMI's impact disaggregated by time period on under-five children fever (Column 1), LLITN utilization among children under-five (Column 2), LLITN ownership (Column 3), and malaria knowledge of respondents (Column 4). Column 1 presents results from a logistic regression model. Columns 2-4 present results from linear regression models. Although not displayed, all models control for survey-round fixed effects. 95% confidence intervals are show in parentheses and are based on Huber's cluster robust variance estimator.

*** p<0.01, ** p<0.05, * p<0.1
